# Understanding landowner preferences for traditional and nature-based solutions incentive programs in North Carolina, USA

**DOI:** 10.1371/journal.pone.0347042

**Published:** 2026-04-13

**Authors:** Courtney B. Deviney, Rajan Parajuli, Frederick W. Cubbage, Erin O. Sills, Robert E. Bardon

**Affiliations:** Department of Forestry and Environmental Resources, North Carolina State University, Raleigh, North Carolina, United States of America; Universitas Airlangga, INDONESIA

## Abstract

Financial incentive programs, commonly administered by public institutions, have long supported sustainable land management practices in the United States, including soil conservation, water quality improvement, and biodiversity preservation. Recently, these initiatives have expanded under the concept of nature-based solutions (NBS), which emphasize land-based practices that deliver co-benefits for people and ecosystems. However, the effectiveness of such programs often depends on how well they align with landowners’ diverse values, preferences, and motivations. This study examines factors influencing forest and farm landowners’ likelihood of enrolling in traditional and NBS-oriented incentive programs. We surveyed 2,000 forest and farm landowners across four regions in North Carolina to assess how ownership motivations, land use intentions, and personal values influence program participation preferences and compensation expectations. The full information maximum likelihood regression results reveal significant differences between forest and farm landowners in their motivations, future land management plans, and financial expectations. Landowners who reside on their land or have never applied to any cost-share programs before are generally less inclined to participate in either program type. These findings highlight the importance of designing targeted outreach strategies and tailoring program structures to better reflect the values and needs of different landowner groups, thereby improving landowners’ participation and enhancing the long-term effectiveness of land-based conservation and sustainability initiatives.

## Introduction

Financial incentive programs have consistently played a pivotal role in advancing sustainable land management, promoting initiatives such as soil conservation, water quality management, and land restoration within the context of natural resource management [1–4]. These programs provide necessary financial support for sustainable forest and farm management to ensure a reliable supply of goods and services while safeguarding biodiversity, soil, and water resources [2,3]. Numerous federal and state programs aim to promote sustainable practices that generate broader public benefits, including clean air and water, alongside wildlife habitat preservation [5]. Typically, these programs operate alongside major legislation such as the Clean Water Act (1972), the Clean Air Act (1970), and the Lacey Amendment (1981), which collectively promote and sustain large-scale conservation and resource management across the United States [6–8].

Financial incentive programs have been widely utilized across the United States to support forestry, agriculture, and other natural resource management efforts. In agriculture, these programs promote sustainable practices such as adaptive crop management, cover cropping, no-till farming, agroforestry, and water conservation [9–11] to enhance soil health, conserve water, and strengthen ecosystem services. Similarly, forestry programs, considering landowner objectives and the characteristics of forestland [12], include management strategies such as site preparation and replanting, thinning, invasive species control, prescribing burning, and infrastructure maintenance. Additionally, ecological enhancement efforts in forestry focus on improving habitat, water regulation, and erosion control, while forest carbon management seeks to optimize carbon sequestration through tree planting and improved forest management [13].

Nature-based solutions (NBS) represent a relatively new concept that integrates natural processes and ecosystem functions to conserve, restore, sustainably utilize, and manage both natural and modified landscapes. NBS address socio-environmental challenges while delivering quantifiable co-benefits for both people and nature [14]. NBS practices often align closely with traditional land management practices in agricultural and forestry contexts such as conservation-oriented agricultural production, enhancement of ecosystem services, establishment of forests, periodic management, and carbon management [14], which are frequently embedded within traditional government incentive programs. These overlaps between forestry, farm and NBS practices underscore how NBS reinforce existing strategies for carbon management, and soil and water conservation.

Landowners’ interests, values, attitudes, and perceptions significantly influence their land management decisions, which consequently affect their participation in financial incentive programs [15]. Previous research showed that landowner attitudes shape engagement in voluntary programs [16] and willingness to accept compensation during environmental and disaster scenarios [17]. Historical programs, such as those introduced in the 1930s to improve agricultural productivity, achieved varying degrees of success depending on their alignment with landowners’ values and beliefs. For example, the Agricultural Appropriation Act of 1933, which diverged from landowner priorities, faced resistance and was eventually defunded, whereas the Soil Bank Program aligned more closely with landowner interests and was sustained and incorporated into subsequent programs [3,18]. Thus, understanding landowner perspectives and values is critical designing effective, acceptable, and sustained conservation programs.

Decision-making processes of landowners related to land management have been examined through frameworks such as the Theory of Planned Behavior (TPB), which integrates social norms, place attachment, and environmental psychology [19–21]. Studies using TPB demonstrated that values, age, and gender significantly influence attitudes and behaviors related to land management [22,23]. Other researchers employed choice experiments and random utility models to investigate how landowners balance personal values and financial incentives in land-use decisions [24,25]. Social norms and stewardship ethics also drive conservation behavior, highlighting the influence of non-monetary motivations [26,27].

Land management decisions are further influenced by economic, environmental, social, and historical factors [15,24]. Economic considerations include costs, land value, return on investment (ROI), and associated financial risks [28,29]. Environmental values incorporate conservation ethics and biodiversity concerns [30,31]. Social factors encompass societal norms, inheritance goals, and family legacy [32,33], while historical factors influence past management practices and familial traditions [34,35].

Research examining land management decision-making frequently integrates these frameworks to evaluate landowner participation in incentive programs. Critical factors include personal motivations, knowledge and prior experiences, perceived incentives, bureaucratic obstacles, and social norms [36,37]. Personal motivations often reflect environmental and historical contexts [38], whereas previous program participation contributes to an individual’s personal history as a relevant component [39,40]. Program awareness depends on the effectiveness of outreach and education [41], while perceived incentives typically involve financial and technical assistance [42–45]. Barriers to engagement often arise from complex application procedures and a lack of trust in governmental agencies [46].

Despite extensive research on landowner behavior, relatively few studies explored its direct interactions with financial incentive programs [17,47–49]. Most focus on conservation easements, overlooking trade-offs associated with active management practices [50]. Program design and regional differences also remain under examined. In North Carolina, for instance, management requirements differ markedly between mountain and Piedmont regions, yet state initiatives rarely account for regional variation [50,51]. Inconsistent participation rates and limited long-term assessments highlight persistent research gaps [51].

A comprehensive understanding of how landowners engage with programs such as the Conservation Stewardship Program (CSP), Environmental Quality Incentives Program (EQIP), and Conservation Reserve Enhancement Program (CREP) is necessary to improve participant engagement and satisfaction. Advances in agricultural practices and evolving forest management techniques may distinctly influence participation, highlighting the need for further examination [39]. The intricate interplay of these factors with the emergence of NBS practices emphasizes the complexity of landowner decision-making and the need for targeted strategies to encourage sustainable engagement.

Despite the availability of numerous financial incentive programs in NC, enrollment remains inconsistent or has declined, suggesting a disconnect between program goals and landowner priorities. Chizmar et al. (2021) [38] identified contributing factors such as limited administrative resources, funding reductions, inconsistent payment structures, and skepticism toward government involvement, all of which hinder participation. Further, many federal and state programs include components for managing both farmland and forestland, thereby introducing administrative complexities for landowners, including time commitments, paperwork, and the need to precisely understand specific requirements [3,52].

This study aims to identify the motivational factors that influence landowner participation in financial incentive programs and to assess how personal values and beliefs affect likeliness to adopt financial incentive programs promoting both conventional and innovative management practices. Additionally, we examine compensation preferences and contract durations to elucidate landowner expectations and motivations for participation in environmental programs. Distinguishing values and preferences across landowners provide insight into barriers to engagement and informs strategies to improve participation.

We contribute to the literature by distinguishing farm and forest landowners to identify variations in their motivations and program participation determinants. This differentiation is crucial for designing tailored policies that enhance landowner participation in sustainable management programs. This research is based on three core hypotheses: (a) personal values (defined as landowners’ reasons for property ownership and their property defined by their reasons for ownership) are significant predictors of participation in financial incentive programs; (b) participation among farm and forest landowners is affected by distinct economic, social, and personal factors; and (c) farm landowners expect higher cost share and annual rental compensation than forest landowners to cover their higher operating costs. Understanding landowner motivations and expectations toward these public incentive programs promoting sustainable land management practices could help program administrators and policymakers design or revise initiatives that foster higher engagement and implementation.

## Data and study methods

### Survey instrument

Data for this study were collected through a mail survey targeting landowners in North Carolina, adhering to the Dillman Tailored Design Method [53]. The 24-question instrument gathered comprehensive information on farm and forest landowners, encompassing social, economic, and ecological factors, as well as their experiences and perspectives on financial incentive programs and their likelihood of participation. The survey took approximately 20 minutes to complete. The target respondents included North Carolina landowners aged 18 years and older who owned at least 10 acres of forest or agricultural land. Large corporate entities (e.g., Weyerhaeuser) and public lands were excluded to focus on non-industrial farm and forest landowners, who represent the majority in the southeastern United States.

The first section of the survey solicited information on land ownership and management practices, particularly property characteristics and management strategies, including acreage, location, ownership duration, land-derived income, current management practices, ownership motivations, and replanting intentions over five-year intervals. To reduce response burden and improve completion rates, we employed a mix of question formats: open-ended items for specific details (e.g., acreage, county), multiple-choice questions with checkboxes (including “other” options), and multiple-response items that allowed participants to select ownership motivations identified in prior literature [36,37,53,54].

The second section of the survey recorded respondents’ opinions and participation in available financial incentive programs. Specifically, respondents were asked whether they had previously enrolled in such programs and whether they would consider future participation. The key participation question was: “If you get an opportunity to participate in a Forestry Incentive Program, Farm Conservation Program, or Nature-based Conservation Program. Would you participate with sufficient incentives?” This question aimed to assess respondents’ general likelihood to engage in financial incentive programs with follow-up questions asking about the rate of initial cost-share reimbursement and annual rental payments required to participate in a 10-year contract. We asked landowners about three program types: farm, forest, and NBS, though detailed practice information was provided only for farm-based and forest-based practices. Based on the definition of NBS described in the introduction and current program brochures, we categorized practices such as site improvement for ecosystem services (e.g., wildlife habitat, water management, erosion control, forest wetland restoration) and ecosystem services (e.g., wildlife cover, stream buffers and restoration, wetlands restoration, runoff/field impoundments) as NBS. While these practices are often included in traditional farm and forestry programs, they were considered representative of emerging NBS practices. Respondents could skip these questions if they were not interested in participating in financial incentive programs. The final section of the survey instrument recorded sociodemographic information, including gender, ethnicity, race, education, income, and age. The survey instrument and the data are included in supporting information (S1 File and S2 File).

### Survey administration

The sampling frame consisted of 180,301 rural landowners identified from the NC OneMap database [55], which compiles parcel data from federal, state, and local sources across North Carolina. The data were filtered using three criteria: a) land size: parcels smaller than 10 acres were excluded; b) ownership type: state-owned, federally owned, and large commercial parcels (e.g., Weyerhaeuser) were removed; and c) land use codes: only properties with county tax codes indicating agricultural, forest, or small-scale rural land uses were retained.

**Fig 1:**
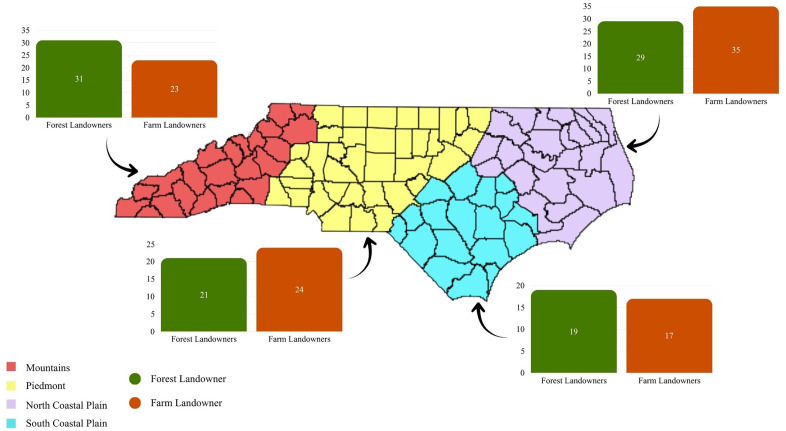
Map of North Carolina (NC) showing counties in each region and response counts by landowner type. Colors represent regional classification, and the accompanying bar graph displays the percentage of responding landowners by region relative to the total surveyed. Republished from NCGS [56] under a CC BY license, with permission from NCDOT, original copyright (2025).

Based on the tax parcel user code delineated for the local assessment by county governments [55], we classified landowners into two categories: forest landowners and agricultural landowners, including both crop and pasture management. To provide regional context, we adopted the USDA Forest Service’s Forest Inventory and Analysis (FIA) program regional classification, dividing the state into Piedmont, North Coastal, South Coastal, and Mountain regions [55]. Using a random number generator in Excel, we selected 250 farm and 250 forest landowners from each region, resulting in a total of 2,000 survey participants. Fig 1 shows the counties included in each region and the distribution of survey responses by landowner type.

Prior to full survey administration, the survey was pretested with eight individuals to evaluate clarity, flow, and usability. Using a modified cognitive interview protocol, participants were observed as they completed the printed survey and were encouraged to provide feedback. Minor revisions to question phrasing and layout were made based on their input. Following pretesting, the finalized instrument was distributed via mail only, consistent with the preferences of North Carolina landowners, who tend to be older [57]. The NC State Institutional Review Board (IRB#25932) reviewed the study design, survey protocol, consent process, and administration procedures, and approved it with exempt.

Survey administration followed the Dillman Tailored Design Method, with four sequential mailings. Survey recipients were initially contacted by mail with an introductory letter, followed by the survey package one week later, a reminder postcard after two weeks, and the second survey copy, in a distinct color, three weeks later. Each respondent received a separate consent form along with the survey instrument and their written informed consent was recorded on the questionnaire. This approach achieved a response rate of 11.9%, yielding 238 responses. Of these,1.8% of the respondents declined consent, resulting in 220 usable responses. Fourteen of the 220 responses were incomplete, which were excluded from further analysis, leaving a final sample of 206 observations for further analysis [58,59].

### Theoretical background for the model

To examine the factors influencing landowners’ likelihood of participation in various land management practices, we developed an empirical model grounded in the willingness-to-accept (WTA) framework, commonly applied in payment for ecosystem services research [60,61]. The basic assumption of WTA is that landowners are willing to participate in these programs if the utility of participating is greater than not participating [62]. In other words, landowners are expected to participate in the programs when perceived benefits exceed opportunity and transaction costs, with decisions shaped by a combination of financial and non-financial motivations [63]. This empirical approach operationalizes the theoretical WTA framework by linking heterogeneous landowner and program characteristics to observed participation outcomes [64]. Let $\overset{p}{\underset{ij}{U}}$ denote the utility landowner *i* obtains from participating in program *j*, and $\overset{np}{\underset{ij}{U}}$ the utility from not participating. Participation occurs if and only if the net gain in utility is positive ($U_{ij}$> 0). This can be formally expressed as:


$$\overset{*}{\underset{ij}{U}}=\overset{p}{\underset{ij}{U}}-\overset{np}{\underset{ij}{U}}$$

where, $\overset{*}{\underset{ij}{U}}$ is the latent (unobserved) net utility of participation. The observed decision rule is therefore to participate in program *j* only if $\overset{p}{\underset{ij}{U}}>\overset{np}{\underset{ij}{U}}$. A landowner accepts the program contract only if the compensation and other perceived benefits are sufficient to offset their opportunity costs, transaction costs, and any disutility from changing land-use practices.

Drawing on existing literature [17,37,51,65,66], we hypothesize that the landowners’ likelihood to participate (LTP) in financial incentive programs is influenced by four categories of factors: economic characteristics including annual income, income derived from property, and ownership motivations related to land investment and management objectives; ecological and land characteristics including current management practices, land location and size, and conservation value; social factors including demographic attributes and property history; and experience factors related to prior enrollment in incentive programs, and additional demographic characteristics. The empirical model is specified as follows:


$$\begin{array}{cc} ogit({PWSI}_{ijg}=\; & \beta \text{0}i+\beta \text{1}iAge+\beta \text{2}iIncome+\beta \text{3}iYearsOwned+\beta \text{4}\text{i}ResideOnLand\\ & \;\;\;+\beta \text{5}iTotalLandAcres+\beta_{\text{6}i}\text{HireAssistance}+\beta_{\text{7}i}\text{SoloManagement}\\ & \;\;\;+\beta_{\text{8}i}\text{ForLandInvestment}+\beta_{\text{9}i}\text{PlantOrReplant5yr}+\beta_{\text{1}\text{0}i}\text{NeverAppliedCostShare}\\ & \;\;\;+\beta_{\text{1}\text{1}i}\text{Mountain}+\varepsilon_{ij}\; \end{array}$$

where :g∈{FOR, FARM, NBS}

The three dependent variables for our models are whether a landowner would participate in forestry programs with sufficient incentives (PWSIFOR), participate in farm programs with sufficient incentives (PWSIFARM), and participate in NBS programs with sufficient incentives (PWSINBS). Each variable is recorded as binary, with 1 being that landowners would participate with sufficient incentives and 0 being they would not. We excluded six “maybe” responses from the analysis because they did not provide sufficient information to interpret respondents’ intentions. Table 1 presents the details of each explanatory variable.

**Table 1:** Description of variables used in the three logistic regression models of landowner likelihood to participate in financial incentive programs.

Variable		Description
Dependent variable		
*PWSIFOR*		Binary variable = 1 if respondent would participate in forestry financial incentive programs with sufficient incentives
*PWSIFARM*		Binary variable = 1 if respondent would participate in farm financial incentive programs with sufficient incentives
*PWSINBS*		Binary variable = 1 if respondent would participate in NBS financial incentive programs with sufficient incentives
Independent variable	*Variable category*	
*Age*	Demographic	Age of landowner, represented by midpoints of categorical responses (24 years, 38 years, 53 years, 68 years, and 80 years)
*Income*	Economic	Approximate total household income in 2020 (recorded in six categories from less than $24,000 to over $150,000)
*Yearsowned*	Experience	Number of years that a landowner has owned their longest-held property in NC
*Resideonland*	Demographic	Binary variable = 1 if respondent resides within 1 mile of their property in NC
*Forestlandowner*	Social/Ecological	Binary variable = 1 if forest is the major land use (over 50% of the total land) of the respondent
*Hireassistance*	Ecological	Binary variable = 1 if landowner hires assistance for any portion of land management
*Forlandinvestment*	Social/Economic	Binary variable = 1 if landowner’s primary ownership motivation is land investment
*Plantoreplant*	Ecological	Binary variable = 1 if respondent plans to plant or reforest any portion of property within the next five years
*Neverappliedcostshare*	Experience	Binary variable = 1 if respondent has never applied for a cost share program.
*Mountain*	Ecological	Binary variable = 1 if most of respondent’s land is located in the Mountain region of North Carolina

Landowners engaged primarily in farming, forestry, or nature-based conservation are likely to face different opportunity costs, management objectives, and institutional constraints, leading to structurally different relationships between their attributes and program participation decisions. However, they are from the same sample and have some relationships between their decisions as the same landowner may own both farm and forestland. Simple linear model may not capture the true effects of some variables which can be different (in terms of magnitude and direction) between the groups.

Since these three equations are interrelated and landowners may consider enrolling the same land in multiple programs, we estimated the three structural equations simultaneously using the Full Information Maximum Likelihood (FIML) approach under a Bernoulli distribution, implemented in STATA’s *gsem* framework [67,68]. This joint estimation approach improves efficiency by accounting for the potential correlation of unobserved factors across program types. In other words, this approach allows each group to have its own parameter vector, thereby explicitly accommodating heterogeneity in how economic, ecological, and social factors influence participation decisions across three program groups. By estimating all equations simultaneously, FIML exploits the full covariance structure of the data, resulting in more statistically efficient parameter estimates than those obtained from separate single-equation models. Moreover, this framework enables valid cross-group comparisons of coefficients and marginal effects, since all parameters are estimated within a unified likelihood function. Conceptually, the model is similar to the segmented or piecewise logistic regression, but FIML improves upon that approach by accounting for interdependence across equations and ensuring consistent and efficient joint estimation. A change in explanatory variables such as income, land size or land ownership motivation is allowed to influence farm, forest and conservation-oriented landowners in different ways. FIML maximizes this joint likelihood over all group-specific parameter vectors simultaneously. This ensures that estimation accounts for heterogeneity across land-use groups while exploiting the full information contained in the data, yielding more efficient and comparable estimates than estimating separate logit models [67].

Additionally, we conducted t-tests to assess whether forest and farm landowners differed significantly in the requested cost-share rates and annual rental payments for various land management practices. Specifically, we evaluated whether the average requested cost-share rates and annual rental payments sought by the two landowner groups for different land use practices were statistically different. For categorical variables (e.g., age, income), midpoint values for each category were assigned to allow their inclusion as continuous predictors in the regression models.

## Results

Of the 206 total responses collected, 63.5% of the respondents were classified as forest landowners, and 33.5% as farm landowners. The remaining 3% owned the equal acres of forest and farmland, so we excluded those responses in further analyses. Responses were received from counties across the state, ranging from one to five or more per county. Table 2 summarizes the various sociodemographic characteristics of respondents by landowner type.

**Table 2:** The frequency distribution of respondents’ socio-demographic characteristics by landowner type, expressed as percentages.

Variable	Category	Forest Landowners	Farm Landowners
N		131	69
*Gender*			
	Female	13.0	30.4
	Male	81.7	69.6
	Other	5.3	0.0
*Income*			
	less than $24,999	0.8	4.3
	$25,000 - $49,999	19.1	7.2
	$50,000 - $74,999	14.5	14.5
	$75,000 - $99,999	15.3	24.6
	$100,000 - $149,999	19.1	15.9
	$150,000 or more	23.7	26.1
*Age*			
	18-30	0.8	0
	30-45	2.3	4.3
	46-60	24.4	21.7
	61-75	50.4	50.7
	75+	21.4	23.2
*Education*			
	High school or GED	9.2	15.9
	Some college degree	21.4	18.8
	Associate’s degree	12.2	15.9
	Bachelor’s degree	26.0	18.8
	Advanced degree	27.5	29.0

Variations in land tenure and property size were observed across ownership categories. Forest landowners had owned their properties for an average of 27.7 years, compared with 32.9 years for farm landowners. The average property size was larger among forest landowners (328.4 acres) than farm landowners (171.4 acres). Regarding participation in financial incentive programs, 45.8% of forest landowners reported prior enrollment in at least one program, compared with 40.6% of farm landowners. The top five motivations for farm landownership included: (1) to pass land to children or grandchildren, (2) agricultural practices, (3) enjoyment of beauty or scenery, (4) privacy, and (5) hunting. For forest landowners, the leading motivations were: (1) enjoyment of beauty and scenery, (2) passing land to children or grandchildren, (3) protecting nature and biodiversity, (4) hunting, and (5) privacy. To provide additional context, Table 3 summarizes the breakdown of forest and farm landowners who reported their likelihood to participate in different types of financial incentive programs. Both landowner types showed their interest in cross-sectoral programs. For instance, about 36.2% of farm landowners reported that they were likely to participate in forestry programs, and about 24% of forest landowners showed interest in farm incentive programs. Overall, forest landowners expressed greater willingness to participate in NBS programs, compared to farm landowners, suggesting broader openness among forest landowners to engage in various conservation-oriented initiatives.

**Table 3:** Count and percentage of forest and farm landowners reporting likelihood of participation across program types.

	Likely to Participate in Forestry Programs	Likely to Participate in Farm Programs	Likely to Participate in NBS Programs
Forest Landowners	57 (43.5%)	31 (23.7%)	51 (38.9%)
Farm Landowners	25 (36.2%)	20 (29%)	19 (27.5%)

Table 4 details the FIML regression results exploring the factors affecting landowner’s likelihood to participate in forestry, farm, and NBS financial incentive programs in North Carolina. With an alpha of 0.1 as a threshold significance criterion, eight factors significantly influenced landowners’ likelihood of participating in forestry incentive programs. Age showed a significant negative association with participation (p = 0.018), indicating that older landowners were less likely to participate in forestry programs. Longer property ownership duration was also negatively related to participation (p = 0.096). Additionally, residing on or near to one’s land was found to reduce the odds of landowners’ participation in forestry incentive programs (p = 0.003), implying that absentee landowners were more likely to enroll. Landowners owning more forestland compared to farmland were more likely to participate in forestry incentive programs. Conversely, hiring management assistance increased participation odds (p = 0.025), as did owning land primarily for investment (p = 0.001) and planning to replant within the next five years (p > 0.001). Landowners who had never previously applied for a cost-share program were less likely to participate (p = 0.003), likely due to limited awareness of available programs.

**Table 4:** Full information maximum likelihood (FIML) regression estimates of factors describing the landowners’ likelihood of participation in forestry, farm, and nature-based solutions programs.

Variables	Forestry (n = 146)		Farm (n = 155)		Nature Based Solutions (n = 153)	
	Odds Ratio (Robust SE)	P > |z|	Odds Ratio (Robust SE)	P > |z|	Odds Ratio (Robust SE)	P > |z|
*Age*	0.931 (0.028)	0.018	0.931 (0.021)	0.001	0.912 (0.023)	>0.001
*Income*	1.140 (0.157)	0.341	1.039 (0.127)	0.756	1.061 (0.011)	0.568
*Yearsowned*	0.982 (0.011)	0.096	0.992 (0.008)	0.304	1.007 (0.003)	0.064
*Resideonland*	0.190 (0.107)	0.003	0.410 (0.187)	0.051	0.329 (0.156)	0.019
*Forestlandowner*	2.244 (0.990)	0.067	0.467 (0.196)	0.070	1.827 (0.746)	0.140
*Hireassistance*	2.760 (1.247)	0.025	1.432 (0.600)	0.391	2.250 (0.893)	0.041
*Forlandinvestment*	5.685 (3.009)	0.001	1.015 (0.471)	0.974	2.078 (0.892)	0.089
*Plantoreplant*	7.820 (4.381)	0.001	1.924 (0.880)	0.152	1.343 (0.577)	0.493
*Neverappliedcostshare*	0.254 (0.115)	0.003	0.250 (0.106)	0.001	0.415 (0.156)	0.019
*Mountain*	0.701 (0.353)	0.480	0.300 (0.138)	0.009	1.055 (0.439)	0.898
_cons	164.091 (347.851)	0.016	336.63 (592.815)	0.001	308.472 (545.181)	0.001

Similar patterns emerged for farm-based financial incentive programs, with five distinct factors showing statistically significant relationship with landowners’ likelihood to participate in farm specific programs. Age (p = 0.001) and residence on or near the property (p = 0.051) both negatively affected the likelihood of participation. Landowners owning more forest land (0.07) and those without prior experience in cost-share programs (p = 0.001) had lower odds, and residing in the mountain region significantly reduced participation likelihood (p = 0.009) of landowners in farm incentive programs.

For NBS programs, six factors were found to be statistically significant explaining landowners’ likelihood of participation in NBS incentive programs. Older respondents (p > 0.001) were less likely to participate in NBS programs. Longer property ownership duration was positively related to participation (p = 0.064). Respondents who hire assistance in managing their land (p = 0.04) and those owning land for land investment (p = 0.089) were positively associated with participation odds. Landowners never applied for a cost-share program in the past decreased their participation odds (p = 0.019).

To examine differences in compensation expectations between forest and farm landowners, we compared the annual per-acre payment requests across program types. Statistical tests were conducted to assess whether the requested payment rates differed significantly between the two landowner groups. Table 5 presents the mean requested initial cost-share reimbursement rates for various practices, along with corresponding *t*-statistics, *p*-values, and indicators of statistical significance.

**Table 5:** Average cost-share reimbursement rates requested per acre by management practice for farm and forest landowners.

Management practice	Forest landowner mean (SE)	Farm landowner mean (SE)	N (Forest | Farm)	T -statistic	P -value
Forest Establishment	52.99 (4.57)	80.28 (14.89)	72|33	−2.26	0.026
Periodic Forest Management	59.40 (4.04)	72.27 (5.31)	72|33	−1.85	0.068
General Maintenance	56.13 (4.67)	63.94 (6.87)	72|33	−0.94	0.350
Site Improvement for Ecosystem Services	61.88 (4.13)	75.61 (5.18)	72|33	−1.95	0.054
Management of Forest Carbon	52.52 (4.58)	68.03 (6.95)	72|33	−1.88	0.063
Farm Production with Conservation Practices	54.81 (5.11)	70.63 (4.89)	53|40	−2.18	0.032
Farm with Ecosystem Services	61.23 (4.88)	66.00 (5.59)	53|40	−0.64	0.522

Forest and farm landowners exhibited significant differences in their average reimbursement requests for five of the seven management practices. Notably, for forest establishment practices, farm landowners requested approximately 30% higher reimbursement rates than forest landowners (p = 0.026), suggesting differing perceptions of costs or benefits associated with establishing and maintaining forest stands. Similarly, farm landowners requested 13% more for periodic forest management (p = 0.068) and approximately 14% more for site improvement for ecosystem services (p = 0.026). Farm landowners also sought 15% higher reimbursements for managing forest carbon (p = 0.063), potentially reflecting differences in familiarity with program structures such as one-time lump sum or contract-based periodic payments or greater awareness of carbon sequestration initiatives within agricultural systems. The final significant variation was observed for farm production with conservation practices, where farm landowners requested roughly 16% (p = 0.032) higher reimbursements, possibly due to the direct benefits of these practices for sustainable agriculture productivity enhancement.

**Fig 2:**
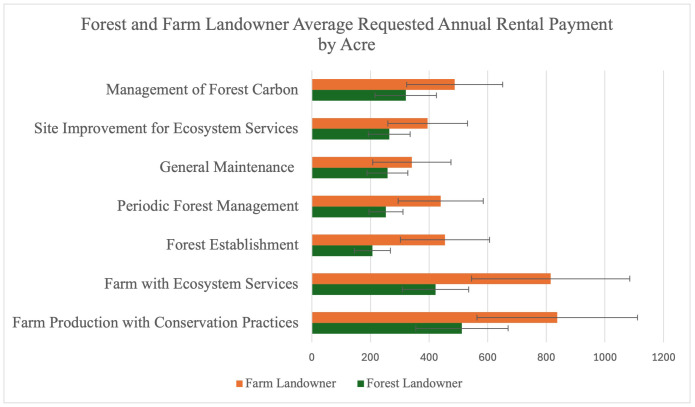
Requested annual rental payments from farm and forest landowners per acre. Black tally lines mark standard errors.

An analysis of annual rental payment differences, illustrated in Fig 2, identified one statistically significant disparity (p = 0.075). Specifically, farm landowners requested an average of $453.88 per acre, compared to $206.61 requested by forest landowners for forest establishment practices. This suggests that farm landowners tend to seek higher rental rates for this practice. Despite the lack of significant differences across most practices, farm landowners consistently requested higher payment rates overall. This pattern indicates that while payment expectations are broadly similar between the two groups, minor variations exist for certain practices, potentially reflecting differing perceptions of opportunity costs or management requirements.

## Discussion

This study highlights the diverse personal values influencing landowners’ engagement in various financial incentive programs. Our findings align with prior studies identifying age, major land use, management strategies, and ownership motivations as significant predictors of participation decisions [40,44,69,70]. A primary finding is that landowners who view their property as an investment are significantly more likely to participate in forestry and NBS programs, while this motivation has no statistically significant effect on the likelihood of enrolling in farm incentive programs. This suggests that indirect economic motivations, such as preserving or enhancing land values, intersect with both financial and non-financial considerations. Additionally, prior enrollment in traditional programs is found to affect landowners’ participation odds, underscoring the importance of past experience with financial incentive programs. On the other hand, distinct value systems were observed between forest and agricultural landowners. Forest landowners are highly likely to enroll in forestry programs but are found to less likely participate in farm programs. Agricultural landowners tended to request higher cost-share reimbursement rates and annual rental payments, reflecting the more intensive and frequent management costs of farming along with higher annual land rental rates [71].

Consistent with previous work [51], we found that privacy concerns negatively influence landowners’ intention to participate in financial incentive programs. Landowners who lived on or near their property were less likely to participate, often citing concerns such as, “I don’t want my land to be accessed whenever the government feels like it,” or “I like my land to look how I like, not how people tell me.” These sentiments reflect broader distrust toward government agencies and fears of property rights restrictions [72]. Further, absentee landowners tend to seek external resources for property management [73], while resident landowners are more inclined to utilize personal resources or alternative methods. Previous studies also suggested that diverse management objectives, personal circumstances, social norms and pressures also shape landowners’ decisions in their land management [74–77]. Future work should consider additional sociocultural and behavioral dimensions to fully understand these dynamics in human dimensions of forest management.

Decision-making among landowners inherently involves uncertainty, as it is impossible to quantify all situational factors. Our findings reaffirm that forest and farm landowners hold distinct yet overlapping values. Notably, farm landowners tend to seek higher compensation than forest landowners, likely to reflect more frequent operational expenses and active management required for farming, which is less prevalent in forest management. However, no significant differences were observed in the rental payment expectations, suggesting that both groups perceive comparable opportunity costs for conservation practices. It may also be attributed to the loss of income from agricultural land to switch to forestry practices. These findings highlight the importance of accounting for the heterogeneity in personal values, costs, and motivations when designing and promoting financial incentive programs.

Results also suggest that younger landowners were more likely to participate in all program types, suggesting greater receptivity to financial incentives or long-term land stewardship goals, in contrast to older landowners who may derive less benefit. This finding aligns with prior research [10,78]. Furthermore, landowners with no prior experience in incentive programs were less likely to enroll in any program types, highlighting the need for targeted outreach and education to first-time applicants, echoing the conclusions of previous studies [40,79]. Collectively, these results underscore that participation in financial incentive programs is shaped by a complex interaction of economic, personal, and structural factors [25,38,50,57,79,80].

Additionally, landowners planning reforestation in the next five years were more likely to engage in forestry incentive programs, likely to offset establishment costs, as noted by [39]. This finding emphasizes the influence of future management intentions on participation behavior, which is an underexplored dimension in prior studies. Farm landowners appeared particularly sensitive to the regional context, with those in the mountain region showing lower participation odds, possibly due to geographic or biophysical constraints. Future research should integrate regional variables to better understand such spatial heterogeneity.

Interestingly, our analysis also revealed that landowners’ requested compensation amounts often exceeded current program reimbursement rates in North Carolina. This misalignment suggests that existing financial incentives may not fully cover perceived costs or opportunity losses. Programs such as the North Carolina Forest Development Program (NCFDP) provide relatively close reimbursement levels, averaging only about $3 less than requested. However, most of the programs are limited by strict acreage and total budget caps, resulting in lower per-acre payments or partial reimbursements. Some respondents provided insights regarding current payment levels, suggesting that landowners in certain regions such as the mountain region of North Carolina already receive higher per acre payments, complicating expectations and emphasizing the need for enhanced communication from program developers regarding payment structures and funding distribution. Enhancing transparency in how payment rates are determined and exploring differentiated payment structures could improve program alignment with landowner expectations. Improved communication regarding payment structures, eligibility criteria, and funding priorities may therefore strengthen trust and participation.

## Conclusion

This study demonstrates that personal and economic values play a critical role in shaping landowners’ participation in financial incentive programs, with clear distinctions between forest and farm landowners. Across all program categories, older and resident landowners were less likely to participate, whereas viewing land as an investment could increase participation likelihood. Farm landowners generally requested higher cost-share reimbursements and annual rental payments than forest landowners, with demographic and regional factors further influencing these preferences. These differences highlight distinct value systems and management priorities across landowner types, underscoring the importance of designing incentive programs that reflect these variations. While cost-share reimbursement expectations differed, no significant differences were observed in rental payment requests, suggesting that landowners perceive one-time and ongoing incentives differently.

Given the overlapping goals of forestry, farming, and NBS, our results imply a readiness for greater integration of NBS practices. We conclude that the principles of nature-based management are primarily aligned with the values and practices that landowners are willing to support, promoting a sustainable future. Future research should collectively investigate the challenges and opportunities inherent in implementing a more integrated NBS approach to land management. Additionally, our findings reveal that forest and farm landowners differ in various aspects beyond ownership. While they may engage in similar land management practices for various objectives, the extent and likelihood of adopting these practices warrant further investigation.

It is important to acknowledge a few limitations that may inform future research. Missing data on program preferences and survey misprints resulted in the exclusion of certain financial incentive variables from regression analyses. These data gaps reflected respondent choices rather than incomplete responses, and imputation was therefore not appropriate. Additionally, out-of-order surveys may have caused some confusion, though their effect on response rates is unclear. Other limitations include potential respondent bias, skepticism toward government programs, and the limited availability of comparable prior studies. In terms of the analysis, future studies should employ tests for measures of association along with measures of significance while comparing two mean values of average cost-share reimbursement rates.

Overall, the results emphasize the need for tailored outreach and program designs that incorporate the diverse motivations and compensation expectations of forest and farm landowners. Addressing these value-based differences can enhance participation and program effectiveness. Future research should examine how awareness, trust, and compensation preferences interact to influence participation decisions and conservation outcomes.

## Supporting information

S1 File Survey instrument used to collect the data. (PDF)

S2 File The dataset used in empirical analysis. (PDF)
