# Supplementary material for: Understanding landowner preferences for traditional and nature-based solutions incentive programs in North Carolina, USA
Source: PLoS One. 2026 Apr 13;21(4):e0347042. doi: 10.1371/journal.pone.0347042 (PMC13075709; doi:10.1371/journal.pone.0347042)
Supplement: S1 File — (PDF) [file pone.0347042.s001.pdf]

Survey Instrument

Section A. General Questions on Land Ownership

1. How much land do **you** own or jointly own in the state of North Carolina and how much of that land is woodland or farm land?

   Total land owned ___________ acres

   Crop land ___________ acres
   Pasture land ___________ acres
   Forest land ___________ acres
   Other (Type: _________________) ___________ acres

2. What was the primary method for acquiring your forestland or farmland in North Carolina? (*please circle all that apply and acreage if multiple*)
   a. Purchased: __________ acres
   b. Inherited: __________ acres
   c. Received as a Gift: __________ acres
   d. Other: (*please specify*): __________ acres

3. In which county in North Carolina do you own most of your farm/forestland?
   ____

4. How long have you owned your land in North Carolina? (*if you own land in multiple parcels, please use the longest owned*)
   __________ years

5. Do you live on or within 1 mile of any of your farm or forest land? (*please circle one*)

   a. Yes
   b. No

6. On average what percentage of your household annual income comes from the rural land that you own in North Carolina?
   __________ %

7. Please select the statement below which best fits the majority of your Farm (crops and pasture) management situation *(please circle one):*

a. I manage and farm the land myself.
b. I hire a consultant/manager to assist with planning but farm the land myself.
c. I lease my land to another person who is my farm manager.
d. Other: Please specify: _____________
e. I do not own any Farm and Agricultural land.

8. Select from below which best fits your forest management situation *(please circle one):*
   a. I perform or supervise all my forest management operations myself.
   b. I hire a consultant to manage and am mostly very hands off.
   c. I hire a consultant or state agency to write a management plan and am very hands on
   d. Other: ____________
   e. I do not own any forest land.

9. What is your main reason(s) for owning rural land? *(please circle all that apply)*

   1. For main source of income
   2. To enjoy beauty or scenery
   3. To pass land on to my children or grandchildren
   4. For privacy
   5. To protect nature and biological diversity
   6. For agricultural practices
   7. For timber products, such as logs or pulpwood
   8. For non-timber forest products, such as firewood, berries, or mushrooms
   9. For land investment
   10. To protect water resources
   11. For hunting
   12. For recreation other than hunting
   13. To help with carbon sequestration or sell offsets.
   14. Other: _______

10. In the past five years, have you (re)planted trees for the purpose of reforestation or afforestation on any portion of your land? (*please circle one*)
    a. Yes
    b. No
11. In the next five years, do you have plans to (re)plant trees on any portion of your land? (*please circle one*)
    a. Yes
    b. No
    c.

Section B. Forestry and Farm Conservation Programs

12. Have you ever applied and/or enrolled in any of the State or Federal financial incentive or cost share programs available in North Carolina ? (*Please check the circle for programs you have applied for and check if you have enrolled)*

State	Applied	Enrolled
NC Forest Development Program (FDP)	○	○
NC Agricultural Cost Share Program (ACSP)	○	○
NC Conservation Reserve Enhancement Program (CREP)	○	○
NC Present Use Value Property Tax Program	○	○
Southern Pine Beetle prevention program	○	○
Other (please specify) ________________________	○	○
Federal		
Environmental Quality Incentives Program (EQIP)	○	○
Conservation Reserve Program (CRP)	○	○
Conservation Stewardship Program (CSP)	○	○
Other (please specify) ________________________	○	○
	Yes	No
Reforestation Tax Incentives (Federal Income Tax Deductions)	○	○

13. Would you consider enrolling or re-enrolling the land you own in North Carolina in any current cost share or incentive program? (*please circle one*)
    a. Yes
    b. No
14. Please use the scale below to rank your overall experience with the State and/or Federal incentive or cost-share programs that you have participated in.
    (*Please circle the number that best corresponds with your experience on a scale of 1 to 5 with 1 being the least satisfied, 2 being somewhat satisfied, 3 being neither satisfied or unsatisfied, 4 being satisfied, and 5 being most satisfied; Skip this question if you have never participated in any programs*)

Program components	State Programs Experience Level of Satisfaction	Federal Programs Experience Level of Satisfaction
	Least <----------> Most	Least <----------> Most

Application deadlines and process	1 2 3 4 5	1 2 3 4 5
Post submission communication and management process	1 2 3 4 5	1 2 3 4 5
Program contact length or requirements	1 2 3 4 5	1 2 3 4 5
Incentives offered	1 2 3 4 5	1 2 3 4 5
Agency communication and support while retrieving required information and submitting application	1 2 3 4 5	1 2 3 4 5
Management deadlines	1 2 3 4 5	1 2 3 4 5
Cost of management with program provided assistance	1 2 3 4 5	1 2 3 4 5
Accessibility to resources (such as information on programs requirements and where to apply)	1 2 3 4 5	1 2 3 4 5
Other(s): (please describe)	1 2 3 4 5	1 2 3 4 5

15. If you get an opportunity to participate in a Forestry Incentive Program, Farm Conservation Program, or Nature-based Conservation Program:

a. Would you participate with sufficient incentives?

	Yes to Any (move to 15b)	Maybe to any (move to 15b)	No to all (move to Q 18)
i. Forestry Incentive program	○	○	○
ii. Farm Conservation Program	○	○	○
iii. Nature based conservation program	○	○	○

b. Which managing entity would you be more likely to participate with? (*Please circle one)*

i. Federally managed

ii. State Managed
iii. Non-governmentally managed
iv. Management entity would not impact my decision to participate.

These programs typically operate on set contact lengths, cover a portion of initial establishment cost, and sometimes offer additional financial assistance for maintenance activities.

In this next question we will go into more detail about your expectations and desires in relation to the financial and technical side of agricultural (farm land) and nature-based conservation programs followed by forest management programs.

There will be two parts to the question, initially we will ask you to report what cost share rate you would require establishing certain practices on your land these practices are typically lump sum cost share payments that assist with the establishment cost and require completion of the practice for set contract term. Then we will ask you to report what annual rental rate you would require maintaining certain practices, these practices typically require annual management and are awarded with annual payouts.

16. In reference to your **rural or farm land** please answer the two sections below. (***If you do not own rural or farm land please move to question 17)***

a. If the government were to offer a ***10-year contract*** *for the practices below, what cost share rate would you require to establish these select practices on your property?*

**Agriculture or conservation practices**	Rate (in percentage) of cost share payment you would require (*In 10% increments, from 0% to 100%)*
Farm production with conservation practices (e.g., cover crop, no till cropping, hardpan breakup, tiling, agroforestry)	%
Ecosystem services (e.g., wildlife cover, stream buffers and restoration, wetlands restoration, runoff / field impoundments)	%
Other(s): (please describe)	%

b. If the government were to offer a ***10-year contract*** *for the practices below, annual rental payment would you require to establish and maintain these practices on your property?*

**Agriculture or conservation practices**	Minimum annual payment amount you would require to maintain practices (in $20 increments, from $0 {none} or greater, e.g. $20 or more)
Farm production with conservation practices (e.g., cover crop, no till cropping, hardpan breakup, tiling, agroforestry)	$
Ecosystem services (e.g., wildlife cover, stream buffers and restoration, wetlands restoration, runoff / field impoundments)	$
Other(s): (please describe)	$

c. Would you be more willing to participate in any of these practices with:

	Yes	No
A shorter contract length	○	○
A longer contract length	○	○

17. In reference to your **forested land** please answer the two sections below. (***If you do not own forested land, please move to question 18)***

*a. If the government were to offer a **10-year contract** for the practices below, what cost share rate would you require to establish these practices on your property?*

**Forest management practices**	Rate (in percentage) of cost share payment you would require (*In 10% increments, from 0% to 100%)*
Forest establishment / tree planting on crop, pasture, or idle agricultural land	%
Periodic forest management (e.g., thinning, invasive species control, prescribed fire)	%
General maintenance (e.g., road maintenance, fire line maintenance, trail maintenance)	%
Site improvement for ecosystem services (e.g., Wildlife habitat, water management, erosion control, forest wetland restoration)	%
Management for forest carbon (e.g., longer rotations, interplanting)	%

*b. If the government were to offer a **10-year contract** for the practices below, what annual rental payment would you require to establish and maintain these practices on your property?*

**Forest management practices**	Minimum annual payment amount you would require to maintain practices (in $20 increments, from $0 {none} or greater, e.g. $20 or more)
Forest establishment / tree planting on crop, pasture, or idle agricultural land	$
Periodic forest management (e.g., thinning, invasive species control, prescribed fire)	$
General maintenance (e.g., road maintenance, fire line maintenance, trail maintenance)	$
Site improvement for ecosystem services (e.g., Wildlife habitat, water management, erosion control, forest wetland restoration)	$
Management for forest carbon (e.g., longer rotations, interplanting)	$

a. Would you be more willing to participate in any of these practices with:

	Yes	No
A shorter contract length	○	○
A longer contract length	○	○

18. If a cost share program you participated in provided a visible display (such as a sign) that you could place at the entrance of your land stating it was sustainably managed by participation in a program, would that make you more likely, less likely, or cause no effect on your participation in the program *(please circle one)*

    a. More likely
    b. Less likely
    c. No effect

Section C. Demographic Attributes

To understand your responses to the previous questions more clearly, we need to know a few things about your background. Remember that your responses are completely anonymous. Neither your name nor your address will be linked to your responses in any way.

19. Please select the gender that best describes you: (*Please circle one)*

    a. Male
    b. Female
    c. Other: _____________
    d. Prefer not to disclose.

20. Are you of Hispanic or Latino origin? (*Please circle one)*

    a. Yes
    b. No

21. Which of the following best describes you? (*Please circle one)*

    a. Asian
    b. Black or African American
    c. Caucasian
    d. Indigenous/Native American or Alaskan
    e. Native Hawaiian or Other Pacific Islander
    f. Two or more races

22. What was your approximate total household income (AGI) in 2022? (*Please circle one)*

a. less than $24,999
b. $25,000 - $49,999
c. $50,000 - $74,999
d. $75,000 - $99,999
e. $100,000 - $149,999
f. $150,000 or more

23. What is the highest level of schooling/education you have completed? (*Please circle one)*

a. Less than 12th Grade.
b. High school or GED
c. Some college
d. Associate degree (2-year degree)
e. Bachelor's degree (4-year degree)
f. Advance degree

24. What is your age in years? (*Please circle one)*

a. Less than 30
b. 31-45
c. 46-60
d. 61-75
e. 76 or more

----End of the survey----
