# Supplementary material for: Understanding landowner preferences for traditional and nature-based solutions incentive programs in North Carolina, USA
Source: PLoS One. 2026 Apr 13;21(4):e0347042. doi: 10.1371/journal.pone.0347042 (PMC13075709; doi:10.1371/journal.pone.0347042)
Supplement: S2 File — (PDF) [file pone.0347042.s002.pdf]

HHincome	Educationl	Region	Yearsowne	resideonla	ForestMan	Mainsourc	Forlandinv	Replanted!	Plantorepla
6	Bachelors	SouthCoas	40	1	2	0	0	1	1
5	Advanced	SouthCoas	40	1	3	0	1	1	0
5	Bachelors	SouthCoas	24	1	2	0	0	0	0
6	Advanced	SouthCoas	7	1	5	0	0	0	0
0	Bachelors	Piedmont	30	1	1	0	1	1	1
5	Bachelors	NorthCoas	40	0	4	0	0	0	0
6	Bachelors	SouthCoas	4	1	1	0	0	0	0
3	Advanced	SouthCoas	50	1	1	0	0	0	1
5	Advanced	Piedmont	11	0	3	0	0	0	1
6	Advanced	SouthCoas	4	0	3	0	1	0	0
5	Advanced	Mountain	28	0	1	0	0	0	0
5	Advanced	NorthCoas	6	1	2	0	1	1	0
2	Some Coll	NorthCoas	30	0	1	0	0	0	0
5	Advanced	NorthCoas	21	0	3	0	1	0	0
6	Associate	NorthCoas	35	0	2	0	1	1	1
4	High Scho	NorthCoas	5	1	1	0	1	0	0
4	Advanced	NorthCoas	132	1	2	0	1	0	1
2	Bachelors	NorthCoas	24	1	5	0	0	0	0
4	Bachelors	SouthCoas	7	0	4	0	0	0	0
4	Advanced	Piedmont	11	0	4	0	1	0	0
6	Advanced	Mountain	13	0	1	0	1	0	1
2	Advanced	NorthCoas	40	1	3	0	1	0	0
6	Advanced	NorthCoas	20	0	3	0	0	1	0
6	Advanced	NorthCoas	100	1	3	1	1	1	1
5	Some Coll	SouthCoas	25	0	1	0	0	0	0
2	Some Coll	SouthCoas	20	0	1	0	0	0	0
3	Associate	NorthCoas	35	1	1	0	1	0	1
6	Bachelors	NorthCoas	30	1	1	1	0	1	1
6	Bachelors	Mountain	13	1	1	1	1	1	1
4	High Scho	Mountain	40	1	1	0	0	0	0
3	Bachelors	Mountain	30	1	3	0	1	0	0
6	Advanced	Mountain	3	1	3	0	1	0	0
6	Advanced	Mountain	2	1	3	0	0	0	0
3	Bachelors	Mountain	25	1	3	0	1	0	1
4	Advanced	Mountain	50	1	4	0	0	0	0
2	Bachelors	Mountain	60	1	1	0	0	0	0
3	Associate	Mountain	41	1	3	0	1	0	0
6	High Scho	Mountain	5	1	1	0	0	0	0
0	Prefer not	Mountain	30	1	3	0	0	0	0
5	Advanced	Piedmont	25	1	3	0	0	0	0
5	Advanced	Piedmont	22	1	4	1	0	0	0
6	Bachelors	SouthCoas	8	0	5	0	0	0	0
1	Some Coll	Piedmont	43	1	5	0	0	0	0
6	Advanced	Piedmont	500	1	1	0	0	0	1
2	Some Coll	Piedmont	20	1	3	1	0	1	0
2	High Scho	SouthCoas	32	1	4	0	1	0	1
0	High Scho	Piedmont	40	1	1	1	0	0	1
2	Some Coll	SouthCoas	15	1	1	0	1	0	1
2	Some Coll	SouthCoas	23	0	1	0	1	0	0
0	Bachelors	SouthCoas	2	1	1	0	0	1	1
4	Associate	Piedmont	40	1	3	0	0	1	1

2	Prefer not	Piedmont	10	1	2	1	0	0	0
2	Associate	Piedmont	24	1	1	0	1	0	1
		Piedmont	35	1	1	0	0	1	0
2	High Scho	SouthCoas	52	1	3	0	1	0	0
2	Some Coll	Piedmont	3	1	4	0	0	0	0
2	Bachelors	SouthCoas	12	1	4	0	0	0	0
4	Bachelors	SouthCoas	4	0	1	0	1	1	1
3	Associate	Piedmont	35	0	3	0	0	1	0
3	Some Coll	SouthCoas	34	1	1	0	1	0	0
5	Associate	Piedmont	40	1	1	0	0	0	0
4	Advanced	SouthCoas	48	0	2	0	0	1	1
6	Advanced	SouthCoas	11	1	3	0	0	1	1
6	High Scho	SouthCoas	25	1	1	0	0	0	0
5	Advanced	SouthCoas	76	1	5	0	0	0	0
6	Advanced	Mountain	30	0	1	0	0	0	1
3	Associate	SouthCoas	2	1	1	0	0	0	1
2	Associate	SouthCoas	30	1	1	0	0	0	0
6	Prefer not	SouthCoas	55	1	1	0	0	1	0
6	Bachelors	Piedmont	3	1	1	0	1	1	1
4	Advanced	SouthCoas	50	1	3	0	0	1	1
0	Bachelors	NorthCoas	30	1	1	1	0	0	0
2	Associate	NorthCoas	35	1	1	0	0	0	0
0	Bachelors	NorthCoas	21	1	3	1	0	0	0
3	High Scho	NorthCoas	7	1	4	0	0	0	0
3	Some Coll	NorthCoas	41	1	3	0	1	1	0
5	Associate	NorthCoas	23	1	1	0	0	0	0
0	Some Coll	SouthCoas	11	0	1	0	0	0	0
4	Advanced	NorthCoas	1	0	1	0	1	0	0
3	Advanced	NorthCoas	54	0	3	0	1	0	1
2	Bachelors	NorthCoas	3	1	4	0	1	0	0
6	Bachelors	NorthCoas	43	0	3	0	1	0	0
3	Some Coll	NorthCoas	55	0	4	0	0	0	0
4	High Scho	NorthCoas	41	0	1	0	0	0	0
0	Some Coll	SouthCoas	14	0	1	0	0	0	0
0	Associate	NorthCoas	2	0	1	0	0	0	1
6	Bachelors	NorthCoas	17	0	1	0	1	0	0
6	Advanced	NorthCoas	30	0	1	0	1	0	0
4	High Scho	NorthCoas	15	1	1	0	0	1	1
3	Associate	NorthCoas	41	0	4	0	0	0	0
6	Advanced	NorthCoas	32	1	1	0	1	0	0
3	Advanced	NorthCoas	67	1	1	0	0	1	1
4	High Scho	NorthCoas	50	1	1	0	0	1	1
3	High Scho	NorthCoas	30	1	4	0	0	0	0
6	High Scho	SouthCoas	7	1	1	0	1	1	1
6	Advanced	SouthCoas	25	0	5	0	0	0	0
4	Bachelors	NorthCoas	50	1	3	1	0	1	1
5	Bachelors	NorthCoas	20	0	4	0	0	0	0
2	Bachelors	NorthCoas	5	1	4	0	0	0	0
0	Advanced	NorthCoas	20	1	1	0	0	0	0
6	Associate	SouthCoas	18	1	1	0	0	0	0
5	High Scho	SouthCoas	20	1	4	0	0	0	0
3	Bachelors	NorthCoas	3	0	1	1	1	0	0

3	Advanced	NorthCoas	18	0	3	0	1	1	0
5	Some Coll	NorthCoas	23	1	1	0	0	0	1
5	Some Coll	NorthCoas	24	1	1	0	0	0	1
5	Some Coll	SouthCoas	5	0	2	0	0	0	0
5	Advanced	Mountain	32	1	1	0	1	0	1
4	High Scho	Piedmont	8	1	1	0	0	0	0
1	Associate	Mountain	50	1	1	0	0	1	0
4	Associate	Mountain	50	1	1	0	0	0	0
5	Bachelors	Mountain	14	1	1	0	1	1	1
4	Some Coll	Mountain	40	1	1	0	0	0	0
6	Some Coll	Piedmont	20	0	4	0	0	0	0
3	Some Coll	Piedmont	30	0	5	0	1	0	0
5	High Scho	Piedmont	7	1	1	0	0	0	0
2	Associate	Piedmont	17	1	4	0	0	0	0
4	Some Coll	Mountain	18	1	1	0	0	0	0
4	Advanced	Piedmont	24	1	4	0	0	0	0
4	Bachelors	Piedmont	21	1	1	0	0	0	0
5	Some Coll	Mountain	19	1	3	0	0	0	0
3	Bachelors	Piedmont	9	1	1	0	1	1	1
6	Associate	Piedmont	5	0	3	0	0	0	1
2	Some Coll	Piedmont	42	1	1	0	0	0	0
4	Bachelors	Piedmont	25	1	5	0	1	0	0
2	High Scho	Mountain	48	1	1	1	0	0	1
2	High Scho	Mountain	55	1	4	1	1	0	0
4	Advanced	Mountain	8	1	1	0	0	0	1
0	Some Coll	Piedmont	17	1	5	0	0	0	0
5	Bachelors	Piedmont	18	1	1	0	0	0	0
6	Advanced	Mountain	44	1	1	0	0	0	1
1	Some Coll	Mountain	58	1	4	0	0	1	1
4	Bachelors	Mountain	20	1	1	0	0	0	0
6	Advanced	Mountain	35	1	1	0	0	1	1
1	Some Coll	Mountain	30	0	1	0	0	0	0
6	Associate	Mountain	33	1	1	0	1	0	0
3	Associate	Mountain	1	1	1	0	0	1	1
3	Associate	Mountain	40	1	1	0	1	0	0
4	Advanced	Mountain	40	1	1	0	0	0	0
6	Advanced	Mountain	20	1	1	0	0	1	1
4	Associate	Mountain	21	1	1	0	0	0	0
4	Bachelors	Mountain	20	1	4	0	0	1	0
5	Bachelors	Mountain	43	1	1	0	1	0	0
2	less than 1	Mountain	30	0	5	0	1	0	0
4	Advanced	Mountain	33	1	1	0	1	0	1
4	Associate	Mountain	25	1	1	0	0	0	0
2	High Scho	Mountain	40	1	1	0	0	0	0
2	Some Coll	Mountain	38	0	4	0	0	0	0
3	Advanced	Mountain	40	1	1	1	0	0	1
5	Some Coll	Mountain	30	1	3	0	1	0	0
6	Bachelors	Mountain	13	1	1	0	0	0	0
4	Associate	Mountain	15	1	3	0	0	0	1
5	Advanced	Mountain	25	1	2	0	0	0	0
4	Advanced	Mountain	27	1	4	0	0	0	0
5	Bachelors	Mountain	25	1	1	1	1	1	1

6	Some Coll	Piedmont	4	0	1	1	1	0	0
5	Some Coll	Piedmont	33	1	3	0	1	0	1
2	Bachelors	Piedmont	61	1	1	1	0	0	0
6	Bachelors	Piedmont	38	1	3	0	0	0	1
4	Advanced	NorthCoas	31	0	1	0	0	0	0
5	High Scho	Piedmont	43	1	1	1	0	0	1
6	Bachelors	Piedmont	25	1	1	0	0	1	1
5	Some Coll	Piedmont	6	0	1	0	1	1	1
5	Bachelors	Mountain	5	0	1	0	0	1	1
4	Bachelors	Mountain	15	0	1	0	0	0	0
4	Some Coll	Piedmont	32	1	2	0	0	0	0
6	Bachelors	Piedmont	11	1	1	0	0	1	1
6	Some Coll	Piedmont	30	1	3	0	0	0	1
5	Bachelors	Piedmont	12	0	1	0	0	0	0
5	Advanced	Piedmont	22	0	1	0	0	0	0
2	High Scho	NorthCoas	36	1	2	0	0	0	0
6	Some Coll	NorthCoas	70	0	1	0	0	1	1
6	Some Coll	NorthCoas	12	0	4	0	1	0	0
2	Prefer not	NorthCoas	50	0	3	0	0	1	1
6	Some Coll	SouthCoas	27	0	4	0	0	0	0
3	Some Coll	NorthCoas	39	1	1	0	0	1	0
6	High Scho	Piedmont	62	0	4	0	0	0	0
3	Some Coll	SouthCoas	74	1	1	0	0	1	1
4	Associate	SouthCoas	6	0	1	0	0	0	0
2	Prefer not	Mountain	20	0	2	0	0	0	0
6	Bachelors	Mountain	20	0	2	0	1	0	0
6	Advanced	Mountain	35	0	1	0	0	0	0
0	Advanced	NorthCoas	30	0	1	0	0	0	0
4	Bachelors	Mountain	19	0	4	0	0	0	0
3	Advanced	NorthCoas	30	0	4	0	0	0	1
3	Advanced	Piedmont	7	0	4	0	1	0	1
2	Advanced	Mountain	3	0	1	0	0	0	0
6	Bachelors	Mountain	22	0	3	0	0	0	0
6	Advanced	Mountain	53	0	1	0	0	0	0
6	Advanced	NorthCoas	4	0	1	0	0	0	0
5	Some Coll	NorthCoas	36	1	1	0	1	1	0
2	Some Coll	Piedmont	1	0	5	0	0	0	0
3	Associate	Mountain	40	0	4	0	0	0	0
2	Some Coll	Piedmont	0	0	4	0	0	0	0
4	Bachelors	NorthCoas	1	0	5	0	0	1	0
3	Bachelors	Mountain	18	0	1	0	0	0	0
5	Bachelors	Piedmont	15	0	3	0	1	0	1
2	Some Coll	NorthCoas	29	0	4	0	0	1	0
6	Bachelors	Mountain	13	0	3	0	0	0	0
3	Associate	Mountain	35	0	4	0	0	0	0
4	Bachelors	Mountain	18	0	3	0	0	0	0
0	Advanced	Mountain	20	0	1	0	0	1	0
0	Advanced	Mountain	26	0	2	0	0	0	1
6	Advanced	Piedmont	33	0	3	0	0	1	0
4	Some Coll	Mountain	3	1	3	0	0	0	0
5	High Scho	Piedmont	2	0	5	0	0	0	1

PWSIFOR	PWSIFAR	PWSINBS	PWSIFOR	PWSIFaR	PWSINBS	CSFPWC	CSFES	CSFAOP	ARFPWC
1	1	1	2	2	2	25	25	25	20
1	0	1	1	0	1	100	100	70	40
1	1	1	1	1	1	100	90		500
1	1	1	1	1	1	10	10	35	700
1	1	1	2	2	2	0	0		0
1	1	1	1	1	1				
1	1	1	2	2	2	50	50	50	500
1	0	1	1	0	1	100	100	63	25
1	1	1	1	2	2	50	100	67	50
1	1	1	2	2	2	100	100	50	1000
1	1	1	1	1	1				
1	0	1	1	0	1				
0	0	1	0	0	1				
1	1	1	2	2	2				
1	0	0	1	0	0	0	100		0
1	1	1	2	2	2	100	100	100	2500
0	0	0	0	0	0				
1	1	1	2	2	2				
1	1	1	2	2	2	0	10	3	0
1	0	1	2	0	2				
1	1	1	2	2	2	0	100	50	0
1	1	1	2	2	1	60	60	30	0
1	1	1	1	1	1				
1	1	1	1	2	2				
1	1	1	2	2	2				
1	0	0	1	0	0				
0	0	0	0	0	0				
1	1	1	1	1	1	75	75	83	80
1	1	1	2	2	2	10	10	5	0
0	0	0	0	0	0				
1	1	1	2	2	2				
1	1	1	1	1	1				
1	0	1	2	0	1				
1	0	1	1	0	1				
0	0	1	0	0	1	50	50		600
1	0	0	2	0	0				
1	0	0	1	0	0				
1	1	1	1	1	1	85	100	61	30
1	1	1	1	1	1	0	0		0
0	0	0	0	0	0				
0	1	0	0	2	0	100	100		1000
0	0	0	0	0	0				
0	0	0	0	0	0				
1	0	1	2	0	1	50	50	34	25
0	0	0	0	0	0				
1	1	1	2	2	2	10	10	20	50
1	1	0	2	2	0	100	100		100
1	1	1	2	2	2				
1	1	1	2	2	2				
0	0	1	0	0	2	100	100	70	40
1	1	1	1	1	1	75	75		250

1	1	1	2	2	2				
1	1	1	1	1	1	80	100	90	90
1	1	1	2	2					
1	0	0	2	0	0				
1	0	1	1	0	1	0	50	50	0
0	0	0	0	0	0				
1	1	1	1	1	1	50	90		1200
1	1	1	1	1	1	30	20	30	500
1	1	1	1	1	1				
0	0	0	0	0	0				
1	0	0	1	0	0				
1	1	1	1	2	1	0	50	25	0
1	1	1	1	1	1	95	95		2500
0	1	0	0	2	0	100	100	50	7000
1	1	1	1	1	1	0	75	67	0
1	1	1	1	2	2	100	100	50	200
0	0	0	0	0	0				
1	0	0	1	0	0				
1	1	1	1	1	1				
1	1	1	1	1	1	50	75		5000
0	0	0	0	0	0				
0	0	0	0	0	0				
1	1	1	1	1	1	100	100	80	60
1	0	0	2	0	0				
1	0	0	1	0	0				
1	1	1	2	2	2	100	100		5800
0	0	0	0	0	0				
1	1	1	1	1	1	90	90	45	0
1	1	1	1	1	1	80	20		5000
1	1	1	2	2	2				
1	1	1	2	2	2	60	60	30	0
0	0	0	0	0	0				
1	0	1	1	0	1	50	50		500
0	0	0	0	0	0				
1	1	1	1	1	1	80	80	80	80
1	0	0	1	0	0				
1	1	1	1	1	1	0	0		4000
1	1	1	1	2	2	80	80	40	0
1	1	1	2	2	2				
0	0	0	0	0	0				
1	1	1	1	1	1	100	100		500
0	0	0	0	0	0				
0	0	0	0	0	0				
1	1	1	2	2	2	50	50	25	2400
0	0	0	0	0	0				
1	1	0	1	1	0	50	80	88	80
0	0	0	0	0	0				
1	0	1	2	0	2				
1	1	1	1	1	1	80	90		1000
0	0	0	0	0	0				
0	0	0	0	0	0				
1	1	1	2	2	2	100	100	89	55

1	1	1	1	1	1	70	70		400
1	0	0	1	0	0				
1	0	0	2	0	0				
1	1	1	1	1	1	70	70	50	30
1	0	0	1	0	0	0	100		
0	0	0	0	0	0				
1	1	1	2	2	2	10	10	5	0
0	0	0	0	0	0				
1	0	1	2	0	2	50	50	75	100
1	0	1	1	0	1				
1	1	1	1	1	1				
0	0	0	0	0	0				
0	0	0	0	0	0				
0	0	0	0	0	0				
0	0	1	0	0	1				
0	1	0	0	1	0	80	0	35	60
0	0	0	0	0	0				
1	0	0	2	0	0				
1	1	1	2	2	2				
1	1	0	1	1	0	50	50	20	3000
1	1	1	2	1	1				
1	1	1	1	1	1	50	50		1000
1	1	0	2	2	0	100	0	30	20
0	0	0	0	0	0				
1	1	1	1	1	1	100	75		6000
0	1	0	0	2	0				
1	0	1	1	0	1				
1	0	1	2	0	2				
0	0	0	0	0	0				
0	0	0	0	0	0				
0	0	0	0	0	0				
0	0	0	0	0	0				
1	1	1	1	1	1	90	90	45	0
1	1	1	2	2	2	80	80		1000
0	0	0	0	0	0				
1	0	0	2	0	0	80	0		1000
1	1	1	2	2	2	100	50	38	0
0	0	0	0	0	0				
0	0	1	0	0	2				
1	1	1	1	1	1	50	50	30	100
0	0	0	0	0	0				
1	1	1	1	1	1	90	90	45	0
1	0	1	2	0	2				
0	0	0	0	0	0				
0	0	0	0	0	0				
1	1	1	1	1	2	80	80	40	0
1	0	1	1	0	1	50	50		500
0	0	0	0	0	0				
1	1	0	2	2	0	50	50	75	100
0	0	1	0	0	1				
1	0	1	2	0	2	80	80		0
1	1	1	1	2	2	100	100		600

1	1	1	2	2	2	80	100	60	40
1	0	1	1	0	1				
1	0	1	2	0	2	70	30	40	60
1	1	1	1	1	1	20	50	48	60
1	1	1	1	1	1				
1	1	0	1	1	0	95	95	73	50
1	1	1	2	2	2	80	80	80	80
1	1	1	2	2	2	50	50	100	200
1	0	1	1	0	1	0	50		0
0	0	0	0	0	0				
1	1	1	1	1	1				
1	1	1	1	1	1	90	10	25	0
0	1	0	0	2	0	50	50	35	20
1	1	1	2	2	2	50	25		500
1	1	1	1	1	1	100	100	50	1200
0	0	0	0	0	0				
1	0	0	1	0	0				
1	0	0	1	0	0				
1	1	1	1	1	1	60	60	30	0
0	0	0	0	0	0				
0	0	0	0	0	0				
0	0	0	0	0	0				
1	1	1	1	1	1	90	90	68	50
1	1	1	2	2	2	100	100	100	100
1	1	1	1	2	2				
1	0	1	2	0	1				
1	1	1	1	1	1	100	100	40	0
1	1	1	1	1	1				
0	0	0	0	0	0	100	0		0
1	1	1	1	1	1	50	50	50	0
1	1	1	1	1	1	80	20	45	40
1	1	1	1	1	1	50	100		1000
1	1	1	2	2	2	0	0		0
1	1	1	2	2	1				
1	1	1	1	1	1	50	100	88	100
0	0	0	0	0	0				
0	0	0	0	0	0				
1	1	1	2	2	2				
0	0	0	0	0	0				
1	1	1	1	1	1				
1	0	0	1	0	0				
1	1	1	2	2	2	0	0		0
1	0	0	2	0	0				
0	0	0	0	0	0				
1	0	0	1	0	0	100	100		30
1	0	1	2	0	2				
1	0	1	2	0	2				
1	0	1	1	0	1				
0	0	1	0	0	1	10	10		200
0	0	0	0	0	0				
1	0	1	1	0	1				

ARFES	ARFAO	CSFE	CSPFM	CSGM	CSSIES	CSMFC	ARFE	ARPFM	ARGM
20	23	30	30	30	30	30	30	30	30
40	70	100	90	80	90	100	184	184	184
500	238	100	100	100	100	100	0	0	0
700	355								
0	0	100	100	100	100	100	0	0	0
500	275	50	50	30	50	50	500	500	250
25	63	100	100	100	100	0	25	25	25
67	67	75	50	25	75	75	120	120	120
1000	550	100	100	100	100	100	1000	1000	1000
		50	50	50	50	50	100	100	100
1000	0	100	30	20	60	50	185	500	200
2500	1300	100	100	100	100	100	0	2600	2500
0	3	0	10	0	10	0	0	0	0
		100	100	100	100	100	200	200	200
100	50	100	100	100	100	100	20	20	20
0	30	0	100	50	80	0	92	92	92
		50	50	50	50	50	500	500	500
100	83	40	60	50	75	50	120	100	160
0	5	10	10	10	10	0	0	0	0
		80	70	50	80	80	0	0	0
		100	100	100	100	100	0	0	0
600	325								
		100	100	100	100	100	100	200	200
		30	30	30	25	30	40	40	20
30	61	0	80	80	80	100	0	40	80
0	0	0	0	0	0	0	0	0	0
5000	1550								
10	34	50	25	25	20	20	100	60	40
800	205	0	0	0	0	10	0	0	0
200	125	100	100	100	100	100	100	100	100
40	70								
260	165	80	100	20	80	80	2000	2000	200

		0	67	67	100	67	0	67	67
90	90								
		50	60	10	0	0	0	0	0
0	20	0	50	50	50	50	0	0	0
1200	635	75	90	90	90	100	0	0	0
500	500	20	20	20	20	20	500	500	500
		20	20	20	20	20	200	200	200
50	25	50	50	50	50	50	140	60	60
2500	1298	95	95	95	95	95	2500	2500	2500
7000	3550								
260	0	100	75	50	75	75	400	20	20
200	150	100	100	100	100	0	200	200	200
1000	1531	55	50	75	65	100	1000	1000	1000
60	80	80	80	90	100	100	60	60	60
		100	100	100	100	100	200	200	200
5800	2950	0	100	100	100	100	0	2400	2400
0	45	90	100	100	90	100	0	0	0
5000	2525								
0	30	50	50	50	80	100	0	0	0
500	275	0	50	50	50	0	0	2000	1000
80	80	80	100	80	100	100	184	184	184
1000	1000								
0	40	100	50	0	50	80	0	0	0
250	238	0	50	50	0	0	0	500	500
2400	1225	90	90	90	90	90	3000	3000	3000
140	88	100	60	60	80	100	200	100	100
		0	0	0	0	0	0	0	0
1000	543	514	100	100	100	100	514	514	600
100	89	100	100	100	100	100	350	20	20

400	235	90	100	90	100	100	500	500	400
		100	100	100	100	100	4000	2000	4000
		10	10	10	10	10	1000	100	100
30	50	70	70	70	70	70	30	30	30
		100	100	75	100	100	0	0	0
0	5	10	10	10	10	10	0	0	0
100	75	50	50	50	50	50	100	100	100
0	35								
		75	75	75	75	75	0	0	0
4000	0	30	30	0	40	0	1000	1500	0
100	300	40	20	30	50	50	0	0	0
0	30	75	75	75	75	75	20	20	20
0	1544	80	100	100	80	100	0	102	0
		0	20	0	10	0	20	20	20
		50	50	50	50	50	0	180	120
0	45	90	90	90	90	90	0	0	0
1000	540	80	60	100	80	100	0	0	0
0		90	80	80	80	80	0	0	0
0	38	50	50	50	50	50	0	0	0
1000	300	50	50	50	50	50	1000	1000	1000
0	45	0	90	0	90	0	90	90	90
		0	0	0	0	0	0	0	0
0	40	75	75	75	75	75	0	0	0
250	213	50	50	50	50	5	200	500	500
100	75	50	75	50	50	50	60	60	100
0	32	80	80	80	80	80	0	0	0
760	390	100	75	0	100	100	700	600	640

20	60	100	80	80	100	100	3000	160	160
40	0	90	100	90	100	0	120	120	100
60	48	30	30	20	50	30	50	20	20
50	73								
80	80	80	80	80	80	80	160	160	160
100	100	50	50	50	50	50	100	100	200
500	138	50	70	70	70	70	0	0	0
		100	100	200	100	100	200	200	200
0	25	0	0	0	0	0	0	0	0
20	35								
300	219	75	75	75	75	75	500	500	500
1200	650	100	100	100	100	100	1200	1000	1000
		50	100	100	100	100	100	100	100
0	30	0	0	0	100	50	100	0	0
40	68	90	90	90	90	0	90	90	90
100	100	100	100	100	100	100	200	200	200
		0	0	0	80	0	0	0	0
0	0	0	0	0	0	0	0	0	0
0	0								
0	30	50	50	50	50	50	0	0	0
40	45	0	50	0	50	0	0	20	0
1000	538	50	50	50	50	50	100	1000	1000
0	0	0	0	0	0	0	0	0	0
100	88	100	100	100	100	100	100	100	100
0	0	0	50	0	0	50	40	40	40
		80	80	80	80	80	80	0	0
		100	100	100	100	0	30	30	30
		50	50	100	50	50	0	0	20
200	105	0	0	0	40	0	0	0	0
		50	50	90	100	50	100	100	140

ARSIES	ARMFC	Agriculture	Yearsowne	age_mid	PWSINBS	PWSIFOR	PWSIfarm	payforassi	hireassista
30	30	40	4	68				1	1
184	184	0	4	68	1	1	0	1	1
0	0	7	3	68	1	1	1	1	1
		99	2	68	1	1	1	0	1
0	0	10	3	68				0	0
		100	4	68	1	1	1	0	1
500	300	5	1	38				0	0
25	2500	0	4	53	1	1	0	0	0
120	120	90	2	68		1		1	1
1000	0	10	1	53				1	1
		0	3	68	1	1	1	0	0
100	100	0	2	68	1	1	0	1	1
		0	3	68	1	0	0	0	0
		600	3	80				1	1
500	2500	700	4	53	0	1	0	1	1
2600	0	30	2	68				0	0
		95	5	80	0	0	0	1	1
		9	3	68				0	0
0	0	12	2	68				0	1
200	200	3	2	53			0	0	0
20	20	69	3	68				0	1
92	92	145	4	68	1			1	1
		100	3	68	1	1	1	1	1
		240	5	68		1		1	1
		0	3	80				0	0
0	0	0	3	80	0	1	0	0	0
		6	4	68	0	0	0	0	0
100	100	260	3	68	1	1	1	0	1
0	0	18	3	53				0	0
		57	4	68	0	0	0	0	0
		50	3	68				1	1
		0	1	38	1	1	1	1	1
0	0	0	1	38	1		0	1	1
0	0	0	3	68	1	1	0	1	1
		22	4	80	1	0	0	0	1
200	200	0	4	80	0		0	0	0
20	20	1	4	68	0	1	0	1	1
60	80	40	2	53	1	1	1	0	0
0	0	14	3	53	1	1	1	1	1
		20	3	68	0	0	0	1	1
		187	3	68	0	0		0	0
		0	2	68	0	0	0	0	0
		13	4	68	0	0	0	0	0
40	20	400	6	80	1		0	0	0
		80	3	68	0	0	0	1	1
0	1000	8	3	53				0	0
100	100	200	4	68	0			0	0
		9	3	53				0	1
		0	3	68				0	0
		20	1	68		0	0	0	1
500	2000	40	4	68	1	1	1	1	1

67	67	20	2	53				1	1
		0	3	53	1	1	1	0	0
		0	4					0	0
0	0	0	4	80	0		0	1	1
0	0	0	1	68	1	1	0	0	0
		6	2	68	0	0	0	0	1
0	0	10	1	53	1	1	1	0	0
500	500	20	4	68	1	1	1	1	1
		12	4	68	1	1	1	0	0
		7	4	68	0	0	0	0	1
200	200	50	4	68	0	1	0	1	1
60	60	0	2	68	1	1		1	1
2500	2500	17	3	53	1	1	1	0	1
		58	4	68	0	0		0	1
20	100	30	3	53	1	1	1	0	1
200	0	13	1	68		1		0	1
		25	3	68	0	0	0	0	1
		130	4	80	0	1	0	0	1
		14	1	53	1	1	1	0	0
1000	5500	7	4	53	1	1	1	1	1
		0	3	68	0	0	0	0	1
		82	4	68	0	0	0	0	0
60	0	173	3	53	1	1	1	1	1
200	200	9	2	68	0		0	0	1
		105	4	80	0	1	0	1	1
2400	2400	34	3	68				0	0
		16	2	80	0	0	0	0	0
0	0	57	0	53	1	1	1	0	1
		52	4	53	1	1	1	1	1
		49	1	68				0	1
0	0	0	4	68				1	1
		24	4	68	0	0	0	0	1
1000	0	40	4	68	1	1	0	0	1
		16	3	80	0	0	0	0	0
184	184	61	1	53	1	1	1	0	1
		40	3	80	0	1	0	0	0
		91	3	80	1	1	1	0	1
0	0	520	3	68		1		0	1
		100	4	68				0	1
		65	3	68	0	0	0	0	1
0	0	40	4	68	1	1	1	0	1
		505	4	68	0	0	0	0	1
		135	3	53	0	0	0	0	1
3000	3000	70	2	80				0	1
		35	3	80	0	0	0	0	1
100	0	770	4	68	0	1	1	1	1
		0	3	80	0	0	0	0	0
0	0	0	2	80			0	0	0
800	0	27	3	80	1	1	1	0	1
		27	3	80	0	0	0	0	1
		40	3	68	0	0	0	0	1
100	220	225	1	38				0	0

500	500	27	3	68	1	1	1	1	1
4000	4000	0	3	53	0	1	0	0	0
100	200	0	3	53	0		0	0	0
30	30	60	2	68	1	1	1	1	1
0	0	0	3	80	0	1	0	0	0
		0	2	53	0	0	0	0	0
0	0	65	4	68				0	0
		0	4	80	0	0	0	0	0
100	100	0	3	53			0	0	0
		0	4	68	1	1	0	0	0
		40	3	68	1	1	1	0	0
		66	3	80	0	0	0	0	1
		0	2	53	0	0	0	0	0
		24	3	68	0	0	0	0	1
		0	3	68	1	0	0	0	0
		51	3	68	0	0	1	0	0
		30	3	68	0	0	0	0	1
0	0	0	3	53	0		0	1	1
		0	2	38				0	0
1000	0	80	2	53	0	1	1	1	1
		0	4	68	1		1	0	0
0	0	30	3	53	1	1	1	0	0
20	20	131	4	68	0			0	0
		0	4	80	0	0	0	0	0
0	0	31	2	53	1	1	1	0	0
		18	3	53	0	0		0	0
20	0	0	3	53	1	1	0	0	0
40	40	0	4	53			0	0	0
		40	4	68	0	0	0	0	0
		5	3	53	0	0	0	0	0
		300	4	68	0	0	0	0	0
		0	3	68	0	0	0	0	0
0	0	25	3	53	1	1	1	0	0
0	0	8	0	53				0	0
		8	4	68	0	0	0	0	0
0	0	0	4	80	0		0	0	0
0	0	30	3	80				0	0
		150	3	68	0	0	0	0	0
		5	3	53		0	0	0	0
1000	1000	0	4	68	1	1	1	0	0
		0	3	80	0	0	0	0	0
90	90	47	3	53	1	1	1	0	0
0	0	0	3	53			0	0	0
		20	4	80	0	0	0	0	0
		0	4	80	0	0	0	0	0
0	0	50	4	24		1	1	0	0
100	0	135	3	68	1	1	0	1	1
		110	3	68	0	0	0	0	0
100	100	35	3	80	0			1	1
		37	3	68	1	0	0	1	1
0	0	140	3	80			0	0	1
600	648	0	3	53		1		0	0

160	3000	48	1	38				0	0
		0	3	68	1	1	0	1	1
120	0	135	4	80			0	0	1
50	50	25	4	68	1	1	1	1	1
		27	3	68	1	1	1	0	1
		672	4	68	0	1	1	0	0
160	160	169	3	53				0	0
100	100	7	2	68				0	0
0	0	5	2	53	1	1	0	0	0
		0	3	68	0	0	0	0	0
200	200	0	3	68	1	1	1	1	1
0	0	1	2	68	1	1	1	0	0
		70	3	53	0	0		1	1
500	500	1	2	80				0	0
1200	1000	50	3	68	1	1	1	0	1
		20	4	68	0	0	0	1	1
100	100	96	4	80	0	1	0	0	0
		167	2	80	0	1	0	0	1
100	200	461	4	80	1	1	1	1	1
		0	3	80	0	0	0	0	0
		0	4	68	0	0	0	0	0
		0	4	80	0	0	0	0	0
90	160	45	4	68	1	1	1	0	1
200	200	30	2	68				0	0
		0	3	68		1		1	1
1000	0	0	3	68	1		0	1	1
0	0	0	4	53	1	1	1	0	0
		32	3	80	1	1	1	0	1
		37	3	80	0	0	0	0	0
0	0	28	3	80	1	1	1	0	1
20	1000	18	2	68	1	1	1	0	0
1000	1000	5	1	68	1	1	1	0	1
0	0	17	3	53				1	1
		14	4	80	1			0	0
100	100	1	1	53	1	1	1	0	0
		100	4	68	0	0	0	0	0
		12	0	53	0	0	0	0	0
		0	4	80				0	0
		0		68	0	0	0	0	0
		0	0	68	1	1	1	0	0
		0	3	68	0	1	0	0	0
40	40	0	3	68				1	1
0	0	70	3	80	0		0	0	1
		1	3	68	0	0	0	1	1
30	0	0	4	80	0	1	0	0	0
		0	3	68			0	1	1
0	0	0	3	53			0	0	0
		0	3	68	1	1	0	1	1
200	0	5	3	68	1	0	0	1	1
		0	1	68	0	0	0	1	1
200	100	10	1	53	1	1	0	0	1

forestlo3	farmlo3	groupLO
1	0	1
1	0	1
1	0	1
0	1	2
1	0	1
0	1	2
1	0	1
1	0	1
0	1	2
1	0	1
1	0	1
1	0	1
1	0	1
0	1	2
1	0	1
1	0	1
0	1	2
0	0	
0	1	2
1	0	1
0	1	2
1	0	1
1	0	1
1	0	1
1	0	1
1	0	1
0	1	2
1	0	1
0	1	2
0	1	2
1	0	1
1	0	1
1	0	1
1	0	1
0	1	2
1	0	1
1	0	1
1	0	1
1	0	1
1	0	1
0	1	2
0	0	
0	1	2
0	1	2
0	1	2
1	0	1
0	1	2
1	0	1
1	0	1
1	0	1
0	1	2

1	0	1
1	0	1
1	0	1
1	0	1
1	0	1
1	0	1
1	0	1
1	0	1
1	0	1
1	0	1
1	0	1
1	0	1
0	1	2
0	1	2
1	0	1
1	0	1
1	0	1
0	1	2
0	1	2
1	0	1
1	0	1
1	0	1
0	1	2
1	0	1
0	1	2
0	1	2
1	0	1
0	1	2
1	0	1
0	1	2
1	0	1
0	1	2
1	0	1
1	0	1
0	1	2
1	0	1
1	0	1
0	1	2
1	0	1
0	1	2
1	0	1
0	1	2
0	1	2
0	1	2
0	1	2
0	1	2
1	0	1
1	0	1
0	1	2
0	1	2
1	1	2
0	1	2

1	0	1
1	0	1
1	0	1
1	0	1
1	0	1
1	0	1
0	1	2
1	0	1
1	0	1
1	0	1
0	1	2
0	1	2
1	0	1
1	0	1
1	0	1
0	1	2
0	1	2
1	0	1
1	1	2
1	1	2
1	0	1
0	0	
0	1	2
1	0	1
0	1	2
0	1	2
1	0	1
1	0	1
0	1	2
1	0	1
1	0	1
1	0	1
0	1	2
0	1	2
1	0	1
1	0	1
1	0	1
0	1	2
1	0	1
1	0	1
0	0	
0	1	2
1	0	1
1	0	1
1	0	1
1	0	1
1	0	1
0	1	2
1	0	1
1	0	1
0	1	2
1	0	1

0	1	2
1	0	1
0	1	2
1	0	1
1	0	1
0	1	2
1	0	1
0	1	2
1	0	1
1	0	1
1	0	1
1	0	1
0	1	2
1	0	1
0	1	2
1	0	1
1	0	1
0	1	2
1	0	1
1	0	1
1	0	1
1	0	1
1	0	1
0	1	2
1	0	1
1	0	1
1	0	1
1	0	1
0	1	2
0	1	2
0	1	2
1	0	1
1	0	1
1	0	1
1	0	1
1	0	1
0	0	
1	0	1
1	1	2
0	0	
1	0	1
1	0	1
1	0	1
1	0	1
1	0	1
1	0	1
1	0	1
1	0	1
1	0	1
1	0	1
0	1	2
